# Graphical Item Maps: providing clearer feedback on professional exam performance

**DOI:** 10.15694/mep.2018.0000116.1

**Published:** 2018-06-01

**Authors:** Mark Beaves, Euan Wallace, Nathan Zoanetti, Patrick Griffin, Margaret Wu

**Affiliations:** 1 Royal Australian and New Zealand College of Obstetricians and Gynaecologists; 2 The Ritchie Centre; 3 Victorian Curriculum and Assessment Authority; 4 Assessment Research Centre; 5 Educational Measurement Solutions

**Keywords:** fetal surveillance education, assessment, assessment feedback, Graphical Item Map, CTG education

## Abstract

This article was migrated. The article was marked as recommended.

**Background**: Structured feedback is an important component of learning and assessment and is highly valued by candidates. Unfortunately, item specific feedback is generally not feasible for high stakes professional assessments due to the high cost of item development and the need to maintain stable assessment performance characteristics. In a high stakes assessment of fetal surveillance knowledge, we sought to use graphical item mapping to allow informative candidate feedback without compromising the item bank.

**Methods**: We developed Graphical Item Maps (GIMs) to display individual candidate performance in the Royal Australian and New Zealand College of Obstetricians and Gynaecologists (RANZCOG) Fetal Surveillance Education Program (FSEP) multiple-choice question assessment. GIMs use item and person parameter estimates from Item Response Theory (IRT) models to map the interaction between a test taker and assessment tasks of varying difficulty.

**Results:** It is both feasible and relatively simple to provide GIMs for individual candidate feedback. Operational examples are presented from the RANZCOG FSEP assessment. This paper demonstrates how test takers and educators might use GIMs as a form of assessment feedback.

**Conclusions:** Graphical Item Maps are a useful and insightful assessment feedback tool for clinical practitioners partaking in a high stakes professional education and assessment program. They might be usefully employed in similar healthcare professional assessments to inform directed learning.

## Introduction

Assessment programs in undergraduate and postgraduate medical education have been developed to meet a variety of purposes, including the maintenance of professional standards, selection of trainees or students, data collection for program evaluation, and informing supplementary instruction ^1, 2^. Each of these objectives has important roles in ensuring that clinicians possess the appropriate knowledge and skills for safe clinical practice. In particular, feedback about an individual’s assessment performance is valuable ^3^, providing personalised guidance for further learning, particularly, but not exclusively, for candidates not meeting minimum standards.

As emphasised by Brown, Race and Smith ^4^ “All assessment .. should allow students to receive feedback on their learning and their performance. Assessment should be a developmental activity”. In essence, informative feedback is not only valued by the examinee, it enriches the assessment itself and encourages self-reflection as a component of continued professional development ^3, 5^. Indeed, while the purpose of summative selection and professional licensure examinations are appropriately focused on yielding a pass or fail decision; whether based on minimum standards or candidate ranking; it is clear that examinees value detailed feedback ^6, 7^. This is particularly the case when they have been unsuccessful ^7, 8^.

Not surprisingly, seeking feedback has been shown to be positively and significantly correlated with achievement on summative medical education assessments ^10^. In the higher education setting the aspects of feedback that learners most value have been identified ^11^. To be useful feedback should be sufficiently detailed, provided sufficiently often and should be provided in a timely fashion. It should be focused on learning rather than marks or the students themselves, linked to the assessment purpose and to criteria, understandable to the recipient, and acted upon to improve work quality or learning.

Additionally, feedback is unlikely to be well received, nor acted upon if it is not from a credible source. Worse still, it may negatively impact performance if delivered inappropriately ^3^. Given the formative nature of these principles it is not surprising that many summative assessment programs in medical education do not meet these ideals ^12, 13^. That said, increasing numbers of professional medical authorities, such as RANZCOG, are recognising the important role of assessment feedback and are looking to implement more effective reporting strategies as an embedded component of their assessment programs.

When reporting performance in a non-clinical summative assessment setting, feedback has generally been limited to one or more of the following: an overall score or grade, an overall rank relative to peer performance, scores or grades for sub-topics or component assessments, and statements of whether the assessment performance represents a pass or fail ^1^. Such feedback does not meet the standards suggested by Gibbs and others ^11^ and is unlikely to assist the individual with their own directed learning. It is possible to do much more, even in the high stakes summative setting.

Item Response Theory (IRT) offers one way of providing enhanced feedback ^14- 16^. IRT models are often used by assessment developers to estimate difficulty parameter values for assessment tasks, referred to as items. These parameters are assumed to be invariant across assessment situations ^17^. A benefit of considering item difficulty in assessment feedback reports is that it can help to identify the sophistication of the knowledge and skills that a given candidate has acquired, or is lacking, in an assessed topic.

Even without IRT, a report that presents a number of scores by specific sub-topics can provide more useful feedback than a single overall score ^18^. The common practice of providing only one raw score across assessment components has been criticised in medical education research ^12, 19^. In terms of identifying strengths and weaknesses however, even sub-topic raw scores are fallible.

Consider for example, a candidate receiving a raw score of 12 out of 19 for a particular sub-topic in a multiple choice examination. If the assessment demanded higher scores in order to be in line with the overall pass standard, this score might be interpreted as pointing to an area of the overall discipline requiring further learning. However, if there is no indication of whether the seven items answered incorrectly were relatively difficult or easy, the information provided may be misleading. The potential cost is that the candidate devotes disproportionate time trying to improve their knowledge in topics in which they are already sufficiently competent, ignoring areas of deficiency. There is scope for more detailed information about each candidate’s performance.

This can be achieved using Graphical Item Maps (GIMs). GIMs are an adaptation of two earlier diagnostic reports: the *kidmap*, described by Wright and colleagues ^20^ and the diagnostic map ( *DIAMAP*) reported by Doig ^21^. The *kidmap* can be produced as standard output using the *Quest* IRM software package ^22^. The distinction between these earlier maps and GIMs is that in a GIM the numerical codes representing items are replaced with the given topic or skill label and are aligned by topic or skill.

Here we report the use of GIMs as a method of candidate feedback in the context of the high-stakes multiple-choice assessment of the RANZCOG FSEP. This is a summative assessment taken by specialist obstetricians, midwives and trainees as one aspect of their knowledge and competency assessment ^16, 23^.

## Methods

This work was undertaken with the approval of the FSEP Steering Committee of RANZCOG. In this section we explain how the structure of a GIM is compiled and interpreted to provide examinees with individualised assessment feedback suitable for informing their future learning.

Test takers’ item response data were analysed with the Rasch model ^24^ to yield item (difficulty) and person (ability) parameter estimates on a common latent trait scale. The unit of measurement for the Rasch scale is logits. The co-location of persons and items on the same scale provides a framework that is well suited to the graphical depiction of relative strengths and weaknesses for test takers of varying abilities. This is particularly the case across content that may span a range of topics and complexity. The estimation of item and person measures is undertaken using the TAM ^25^ package in the R ^26^ statistical programming language. The measures (logits) derived from the Rasch scale analyses are classified using a number of variables that describe the candidates and assessment items.

**Figure 1.**
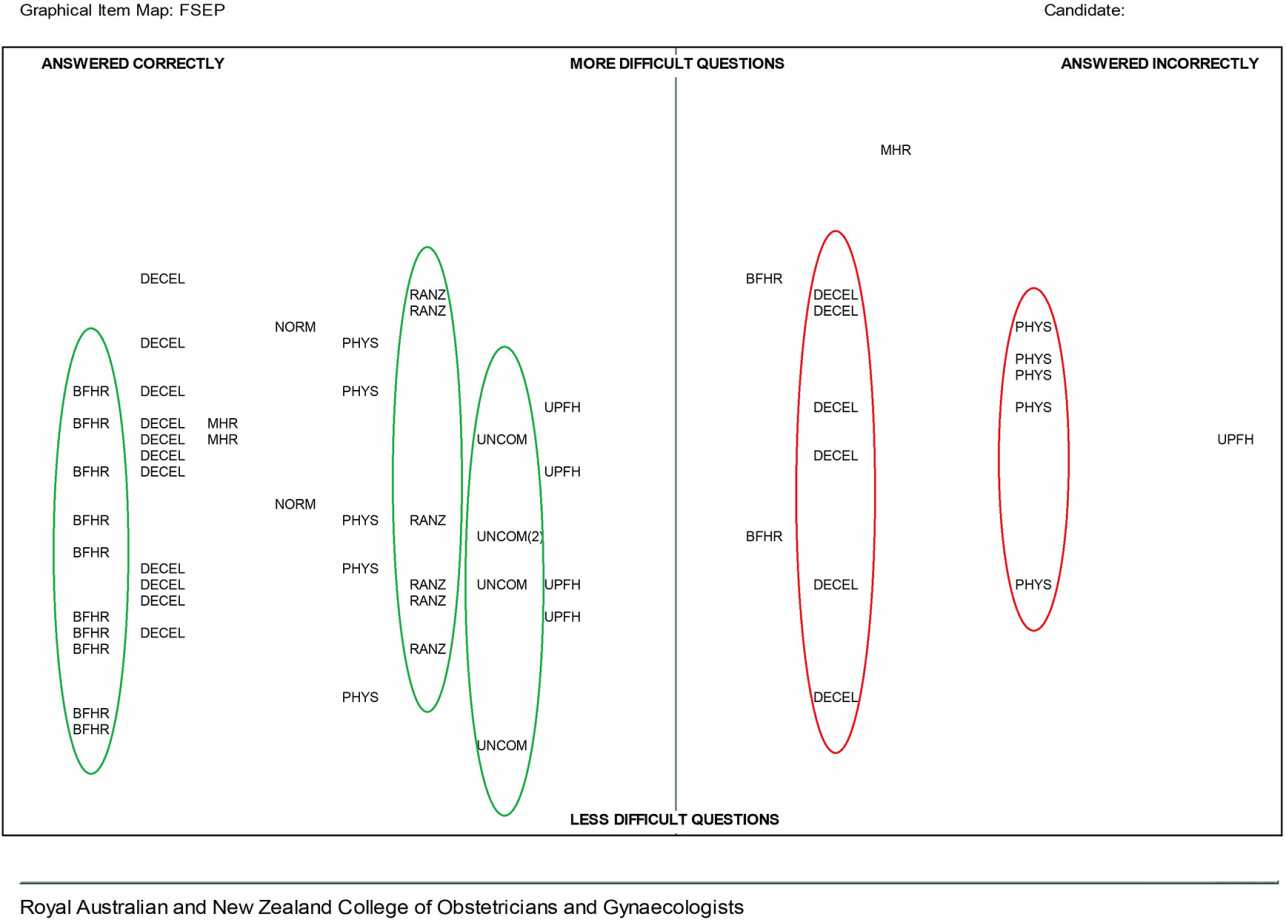
FSEP GIM example 1

To construct a GIM, four variables are used to create the map: *items* (individual questions), *subject cluster,relative difficulty* and *answer key* (correct/incorrect) ( figure 1). There are 60 *items* (questions) in each assessment. The item bank is developed by the FSEP Assessment subcommittee and the statistical performance of every item in use is reviewed annually. The *subject cluster* groups individual items into knowledge domains *,* with each item assigned to one of various topic/skill categories to signify distinct content domain or item typology. The *subject* clusters and their respective abbreviations are:

• the physiology of fetal heart rate control (PHYS **)**
• utero-placental function, including hyperstimulation (UPFH)
• the “normal” CTG as per the RANZCOG IFS Clinical Guideline 2014 ^27^ (NORM)
• the baseline fetal heart rate and baseline variability (BFHR)
• decelerations, including definitions, physiology and management (DECEL)
• maternal heart rate recording (MHR)
• uncommon CTG patterns (i.e. arrhythmias, sinusoidal patterns) (UNCOM) and
• the RANZCOG IFS Clinical Guideline 2014 ^27^ (RANZ).

As described, the estimate of *relative difficulty* for each item is measured in logits from Rasch scaling and graphed vertically. The higher the logit value the more difficult the item. The *Answer key* contains the numbered code corresponding to the correct answer for each item. Candidate ability estimates and assessment pass standards (both measured in logits) can also be recorded for the production of GIMs.

Construction of the GIM then proceeds as follows. For each candidate’s assessment, the individual item (question) is positioned either to the left of the vertical axis if correctly answered, or to the right if incorrectly answered ( figure 1). The items are then mapped into columns with each column representing a *subject.* The vertical position of any given item in the column represents their relative difficulty. Within the *subject* column items with similar difficulty estimates are co-located as a cluster. The number of items within any such cluster is placed in parentheses after the item description.

It is also possible to include the candidate’s IRT score, represented by a horizontal line across the vertical latent scale axis on the map, and the overall pass standard with a similar horizontal line (not shown). Such additions could be used to effectively divide the GIM into four quadrants representing the pass/fail dichotomy intersecting with the item correct/item incorrect dichotomy.

## Results

**Figure 2.**
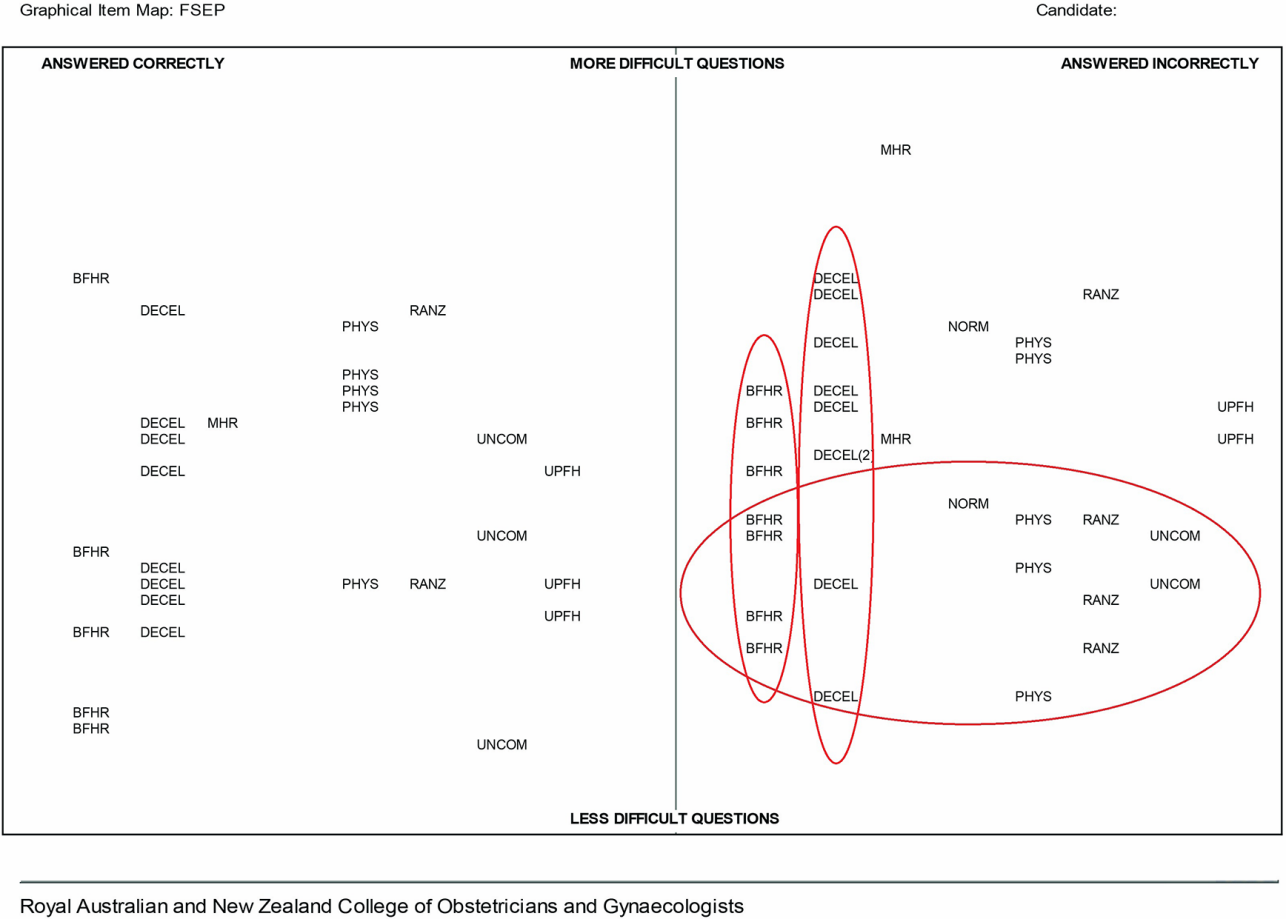
FSEP GIM example 2

**Figure 3.**
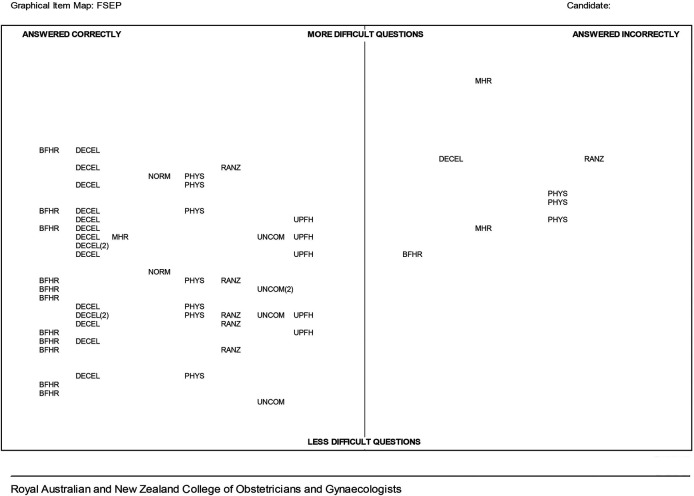
FSEP GIM example 3

Example GIMs from the RANZCOG FSEP multiple-choice assessment, administered as part of the education program ^16, 23^ are shown in the figures 1-3. A GIM is provided to each candidate within two weeks of completion of the assessment, in accordance with recommendations that feedback be detailed and timely ^11^. Candidates are also provided with an explanation of how to interpret and make use of the information embedded in their individualised GIM ( Appendix 1). Additional detailed explanation is is made available on the FSEP website at www.fsep.edu.au ^28^

Figure 1 shows a GIM for a candidate for whom a profile of potential strengths and weaknesses are identified and marked with ellipses. The ellipses; green for potential strengths and red for areas which may require revision, are designed to assist the candidate in targeting their future learning and are explicitly linked to topic headings in the FSEP online course and supporting reference materials ^27- 29^. While strengths are clear for the baseline fetal heart rate/baseline variability (BFHR), the RANZCOG Guideline (RANZ) and the uncommon CTGs (UNCOM), there are potential areas of weakness in decelerations (DECEL) and physiology (PHYS).

In figure 2, a different profile of strengths and weaknesses for another candidate is evident. In this case the topics requiring most urgent attention are baseline fetal heart rate/baseline variability (BFHR) and fetal heart rate decelerations (DECEL). Also apparent is a cluster of relatively simple items which are incorrect. This is a fairly common feature, particularly where candidates are performing below expectations and in our experience, where test anxiety ^30^ may be impacting on candidate performance.

Figure 3 shows a GIM from a candidate who performed well and consequently, there are only eight items on the right (incorrect) side of the map. Further, each of the incorrect items has relatively high logit values and are therefore more difficult items. Additionally they are spread across the map suggesting that this candidate has no specific *subject* area of weakness.

## Discussion

In the paper we have described the development and application of graphical item maps (GIMs) as a feedback tool to healthcare practitioners sitting the College’s FSEP assessment. We show that a graphical display of a candidate’s performance in a manner that can direct future learning is possible without compromising the security of the assessment itself. Specifically, directed feedback highlighting a candidate’s subject weakness and overall performance is possible without revealing the answers to individual questions. We believe that in this high stakes professional assessment, GIMs will become a very useful part of candidate feedback and support, particularly to the weaker candidate.

We believe that the interpretation of the GIMs follows intuitively from the way the items are distributed. Items towards the top of the map are relatively more difficult than those placed near the bottom of the GIM. It also ensures that the information is readily accessible by candidates to direct their future learning. For candidates who have not achieved a given pass standard, the most prudent course of action would be to focus on topics for which there are a number of incorrect items below the pass standard or towards the bottom of the map. These items require knowledge and skills that could be easier to grasp, which corresponds with Gibbs’ point that feedback should focus on learning, not marks or the students ^11^. Focusing on these items can make the learning and examination preparation process more efficient and increases the likelihood of passing the examination on a subsequent attempt ^31^. By identifying topic areas that contain lower-right quadrant items, candidates are provided with a clear starting point for self-directed learning.

Such directed learning support is not only useful for candidates who fail to achieve a required score on the assessment. As a component of enhanced risk management, many hospitals in Australia and New Zealand now require individual practitioners to achieve a particular score or RANZCOG FSEP ‘practitioner level’ ^16^ appropriate for their clinical role. For example, in some hospitals the senior midwifes and the consultant obstetricians on birth suite are required to attain a level 3 practitioner score (>75%) on their FSEP assessment, whereas a student midwife or new resident might initially be expected to attain level 1 (55-65%).

As such, it is possible for an individual to score reasonably well on the assessment but not necessarily achieve the practitioner level required of them. Generally they are then directed to re-sit the FSEP assessment, hopefully with support and after targeted education, as guided by their individual GIM. In this regard the GIM offers the individual guidance on the topic(s) that most merit their attention. As such the FSEP meets the widely accepted requirements of a high quality education program in the provision of timely and structured assessment feedback.

GIMs are also able to provide educators and managers with insights into the overall performance of candidate cohorts and so direct teaching to common areas of weakness. In our experience, selected hospital leadership personnel are increasingly being asked to undertake face-to-face consultations with some of their candidates to discuss potential areas of weakness and appropriate courses of action, based on their assessment score and GIM.

While the GIM and GIM explanation resources are provided by RANZCOG within 2 weeks of attending a program; a limitation not considered in this paper is the additional resources potentially required by a hospital to provide additional feedback and guidance to their clinicians not achieving a required level or score. In our experience the information provided by the GIM appears particularly useful to candidates whose performance is near the pass standard or required practitioner level, with only one or two areas needing improvement. The graphical report on their performance provides them with an efficient path to overcome specific knowledge or skill deficiencies and enhance their chances of meeting their required pass standards.

Of course, it is possible that GIM feedback may impair future learning. Indeed it has been shown by others that feedback *per se*, whether positive or negative, may be detrimental to subsequent performance ^32^ In that regard, in the setting of providing feedback on an individual’s performance in an assessment, particularly on underperformance in a high stakes assessment, it is important to avoid detrimental effects.

Regulatory focus theory ^33^ attempts to describe why feedback, even positive feedback, may be detrimental to future learning. However, according to that theory we believe that the visualisation afforded by GIM feedback would enhance future learning of the poor scoring candidate by being motivational under prevention focus - essentially showing the candidate what he/she must do to pass. Assessing whether regulatory focus theory applies to the feedback afforded by the GIM, and therefore the effectiveness of GIMs in supporting poorly performing candidates, would certainly be worthwhile.

Another limitation of the GIMs presented in this paper is that they do not provide item- specific content feedback to candidates. It has been suggested that candidates sitting multiple-choice assessments benefit when the correct answers and a discussion of the answers are provided to them immediately after the examination ^34^. However, the cost of developing and maintaining banks of items that perform with the validity and reliability required of high stakes assessments is considerable. Therefore, providing details of items in the public domain is unlikely to be a feasible option for most medical education providers ^15^. This is especially the case where the assessment is geared towards high-stakes decisions directly related to an individual’s employment status, like the RANZCOG FSEP assessment. This might also be the case where the stability of item properties, like relative difficulty, is being relied upon to maintain pass standards over time ^17^. Nonetheless, even under the constraint of needing to keep the test items secure, GIMs convey significantly more information than simply an individual’s score. Importantly, by focusing on skill and content areas rather than specific items, GIM feedback does not explicitly encourage ‘teaching to the test’ but rather supports directed content learning.

Further enhancement of the GIMs could include described proficiency levels ^24^. These define the generalised levels of knowledge represented by the test items. This would usefully add to the current report by describing in detail the nature of the developmental skills and knowledge embedded in clusters of items. This might in turn assist the candidate in identifying the domain of knowledge as well as the level of sophistication to aim for in future revision.

Another GIM enhancement might include depiction of performances on complex assessment tasks that apply partial credit scoring. In such a case each score category within a partial credit item could be mapped according to its relative difficulty and whether or not it was attained. For example, if a candidate scored two out of a possible three score points for an assessment task the map would display the relative difficulties of score categories one and two on the left and the relative difficulty of score category three on the right.

This extension would be feasible for assessments analysed with a Rasch Partial Credit Model ^35^. While this would be an extension of current graphical item mapping, it is also possible to simplify it using Classical Test Theory (CTT) instead of Rasch modelling, because GIMs do not require Rasch measurement for the scaling of assessment data. Instead, a GIM could be produced using indices such as facility (the percentage of correct respondents for an item). Items could simply be positioned along the vertical facility axis that would be expressed as a percentage from 0 to 100. This would likely make GIM-like reporting feasible for more assessment programs.

One important caution regarding the use of GIMs for candidate feedback is the reliability and validity of the assessment being reported on. The accuracy and reproducibility of the interpretation made from a GIM would be questionable if the assessment was unreliable or contained poorly constructed test items. Guidelines for omitting test items on statistical grounds therefore might be a useful component of GIM production policy. In this regard the reliability and validity of RANZCOG FSEP items are formally reported externally and critically assessed annually ^16^ by the assessment subcommittee of the program, allowing under performing items to be amended or replaced.

In conclusion, GIMs are presented as an assessment feedback tool that is useful to candidates in ways that a combination of scores, grades or ranks is not. Providing candidates with GIM reports upholds the ideal that assessment should support learning and professional development and thereby enhance the performance of the workforce more generally.

## Take Home Messages

• All assessment should allow students to receive feedback on their learning and their performance.
• Structured feedback is an important component of learning and assessment and is highly valued by candidates.
• Informative feedback is valued by the examinee and enriches the assessment itself.
• GIMs are an assessment feedback tool which may be useful to candidates in ways that a combination of scores, grades or ranks is not.

## Notes On Contributors

Mark Beaves is a midwife, PhD student at Monash University and manager of the RANZCOG Fetal Surveillance Education Program.

Professor Euan M Wallace is the Carl Wood Professor of Obstetrics and Gynaecology at Monash University and the CEO of Safer Care Victoria.

Professor Patrick Griffin is the Emeritus Professor at the Assessment Research Centre, University of Melbourne Graduate School of Education.

Professor Margaret Wu is an expert in educational measurement.

Dr Nathan Zoanetti is an expert educational measurement practitioner. N. Zoanetti was involved in this work when previously associated with the Assessment Research Centre, University of Melbourne Graduate School of Education. The views expressed in this article do not necessarily reflect the views of the Victorian Curriculum and Assessment Authority.

## Appendices

### Appendix 1. FSEP Graphical Item Map explanation, legend and map, as received by all test takers

**Table:**

**GRAPHICAL ITEM MAP EXPLANATION AND LEGEND **

The individual Graphical Item Map you have received is designed to help you identify your strengths and potential weaknesses in fetal surveillance, as determined from the FSEP test you recently undertook.

Questions (items) in our test are ranked from easiest at the bottom of the map, to hardest at the top. This information is derived from our previous extensive and ongoing testing and assessment of these individual items.

Items answered correctly are to the left of the map and those answered incorrectly are to the right of the map.

The presence of brackets following an item i.e. Phys (2), indicates multiple items at that position, with the same degree of difficulty.

The items are grouped into broad subject headings. They are labelled in this way to guide you to the educational resource that will provide you with the information necessary to answer questions within that subject heading correctly. This could include the face to face FSEP sessions, our book Assessing fetal wellbeing: a practical guide (2016), one of the Online Fetal Surveillance Education Programs (OFSEP or OFSEPlus) or the RANZCOG Intrapartum Fetal Surveillance Guideline (2014).

The subject headings are:

**Table:**

PHYS	The physiology of fetal heart rate control
UPFH	Utero placental function, including hyperstimulation
NORM	Features and definitions of the “normal” CTG per the RANZCOG Guidelines
BFHR	Baseline fetal heart rate and baseline variability (normal and abnormal)
DECEL	Decelerations, including the physiology, definitions and management
MHR	Maternal heart rate recording
UNCOM	Uncommon CTG patterns i.e. arrhythmias, sinusoidal patterns
RANZ	The RANZCOG Guidelines i.e. indications for EFM, definitions

If the majority of items in a given subject are grouped to the left of the map (answered correctly) this is an area you have covered well in your study. If items are clustered to the right of the map (answered incorrectly) this is an area you may wish to focus on in future study. This is especially true if these items are also close to the bottom of the map.

Being able to identify your strengths and weaknesses this way will allow targeted education to improve your knowledge, clinical skills and test performance.

In the sample Graphical Item Map below, the areas which have been well covered and answered correctly are the normal CTG, the physiology of fetal heart rate control and the RANZCOG Guidelines. Areas covered less well are the baseline fetal heart rate and fetal heart rate decelerations. In the sample case below, the inability of the candidate to correctly identify the baseline fetal heart rate on a CTG also reduces their chances of correctly identifying any given deceleration.

Concentrated effort on properly identifying the baseline fetal heart rate should improve the practitioner’s knowledge, clinical skills and test performance.

## References

[1] McLachlan JC Whiten SC . Marks, scores and grades: scaling and aggregating student assessment outcomes. Med Educat. 2000;34:788–97 10.1046/j.1365-2923.2000.00664.x 11012927

[2] Fowell SL Maudsley G Maguire P Leinster SJ Bligh J . Student assessment in undergraduate medical education in the United Kingdom. Med Educat. 2000;34(Suppl 1):1–49. 10.1046/j.1365-2923.2000.0340s1001.x 11016480

[3] MacKinnon MM . Using observational feedback to promote academic development. Int J Acad Dev. 2001;6:21–28 10.1080/13601440110033689

[4] Branch WT Paranjape A . Feedback and reflection: teaching methods for clinical settings. Acad Med. 2002;77:1185–8 10.1097/00001888-200212000-00005 12480619

[5] Brown S Race P Smith B . 500 Tips on Assessment. Kogan Page, London,1996

[6] Duffield KE Spencer JA . A survey of medical students’ views about the purposes and fairness of assessment. Med Educat. 2002;36:879–886 10.1046/j.1365-2923.2002.01291.x 12354251

[7] Fox S Reid WA Evans P . Web-based feedback of medical student assessment results. Med Educat. 2003;37:1036–7 10.1046/j.1365-2923.2003.01645.x 14629431

[8] Dillon GF Marcus LA Walsh WP . The usefulness of test-performance feedback in preparing to repeat the USMLE Step 3 Examination. Acad Med. 1997;72:S94–6. 10.1097/00001888-199710001-00032 9347752

[9] American Educational Research Association, American Psychological Association, National Council on Measurement in Education . Standards for educational and psychological testing. Washington, DC: APA, AERA, NCME,1999

[10] Sinclair HK Cleland JA . Undergraduate medical students: who seeks formative feedback? Med Educat. 2007;41:580–582 10.1111/j.1365-2923.2007.02768.x 17518838

[11] Gibbs G . How assessment frames student learning.In: Innovative Assessment in Higher Education. Bryan C Clegg K. (eds). Routledge, New York,2007. pp23–36

[12] Fowell SL Jolly B . Combining marks, scores and grades. Reviewing common practices reveals some bad habits. Med Educat. 2000;34;785–786 10.1046/j.1365-2923.2000.00796.x 11012925

[13] Fowell SL Southgate LJ Bligh JG : Evaluating assessment: the missing link? Medic Educat. 1999;33:276–281 10.1046/j.1365-2923.1999.00405.x 10336758

[14] McManus IC Thompson M Mollon J . Assessment of examiner leniency and stringency (‘hawk-dove effect’) in the MRCP (UK) clinical examination (PACES) using multi-facet Rasch modelling. BMC Medical Education. 2006;6:42 10.1186/1472-6920-6-42 16919156 PMC1569374

[15] Roberts C Zoanetti N Rothnie I . Validating a multiple mini-interview question bank assessing entry-level reasoning skills in candidates for graduate-entry medicine and dentistry programs. Med Educat. 2009;43:350–359 10.1111/j.1365-2923.2009.03292.x 19335577

[16] Zoanetti N Griffin P Beaves M Wallace EM . Rasch scaling procedures for informing development of a valid Fetal Surveillance Education Program multiple-choice assessment. BMC Medical Education. 2009;9:20. 10.1186/1472-6920-9-20 19402898 PMC2685791

[17] Downing SM . Item response theory: applications of modern test theory in medical education. Med Educat. 2003;37:739–745. 10.1046/j.1365-2923.2003.01587.x 12945568

[18] Leonie-Perkins ML Dillon GF Walsh WP . Examinee perceptions of the usefulness of performance feedback on an examination for medical licensure. Academic Medicine. 1996;71:S88–90. 10.1097/00001888-199610000-00054 8940945

[19] Wilson I . Combining assessment scores: a variable feast. Medical Teacher. 2008;30:428–430 10.1080/01421590802043843 18569667

[20] Wright BD Mead RJ Ludlow LH . KIDMAP: Person-by-Item Interaction Mapping. MESA Memorandum. MESA Press, Chicago 1980.

[21] Doig B . DIAMAPs - Self-scoring kidmaps. AERA Annual Conference. 1992, San Francisco (ERIC Document Reproduction Service No. ED346146).

[22] Adams RJ Khoo S-T . Quest: the interactive test analysis system, Version 2. The Australian Council for Educational Research, Camberwell,1996.

[23] Zoanetti N Beaves M Griffin P Wallace EM . Fixed or mixed: a comparison of three, four, and mixed-option multiple-choice tests in a Fetal Surveillance Education Program. BMC Medical Education. 2013;13:35 10.1186/1472-6920-13-35 23453056 PMC3599143

[24] Bond T & Fox CM . Applying the Rasch model: Fundamental measurement in the human sciences. 2001 L Erabaum associates. NJ

[25] Robitzsch A. Kiefer T. & Wu M. (2017). TAM: Test analysis modules. R package version 2.4-9. https://CRAN.R-project.org/package=TAM

[26] R Core Team (2017). R: A language and environment for statistical computing. R Foundation for Statistical Computing. Vienna, Austria. ISBN 3-900051-07-0, URL http://www.R-project.org/

[27] RANZCOG . Intrapartum fetal surveillance Clinical Guideline Third edition. 2014.

[28] RANZCOG FSEP online programs (OFSEP and OFSEPlus).

[29] Baker L Beaves MC Wallace EM . Assessing fetal wellbeing: a practical guide. RANZCOG and Monash Health, Melbourne,2016

[30] Sarason I.G . Test anxiety and intellectual performance. The Journal of Abnormal and Social Psychology. 1963;66:73–75 13991467 10.1037/h0047059

[31] Griffin P . The comfort of competence and the uncertainty of assessment. Studies in Educational Evaluation. 2007;33:87–99 10.1016/j.stueduc.2007.01.007

[32] Kluger AN DeNisi A . The effects of feedback interventions on performance: a historical review, a meta-analysis, and a preliminary feedback intervention theory. Psychol Bull. 1996;119:254–284 10.1037/0033-2909.119.2.254

[33] Watling C Driessen E Van Der Vleuten CPM Vanstone M Lingard L . Understanding responses to feedback: the potential and limitations of regulatory focus theory. Med Educ. 2012;46:593–603 10.1111/j.1365-2923.2012.04209.x 22626051

[34] Larsen DP Butler AC Roediger HL . Test-enhanced learning in medical education. Medical Education. 2008;42:959–66. 10.1111/j.1365-2923.2008.03124.x 18823514

[35] Masters G . A rasch model for partial credit scoring. Psychometrika. 1982;47:149–174 10.1007/BF02296272

