# Supplementary material for: Graphical Item Maps: providing clearer feedback on professional exam performance
Source: MedEdPublish (2016). 2018 Jun 1;7:116. [Version 1] doi: 10.15694/mep.2018.0000116.1 (PMC10699391; doi:10.15694/mep.2018.0000116.1)
Supplement: Peer review history

# Reviewer report: Reviewer response for version 1

**DOI:** 10.21956/mep.19583.r29067

**Published:** 2018-06-21

**Authors:** P Ravi Shankar

**Affiliations:** 1 American International Medical University

This review has been migrated. The reviewer awarded 4 stars out of 5 The authors describe an interesting approach to provide feedback on student performance. Graphical item maps (GIMs) from the authors’ description seem to be effective in providing simple yet directed feedback to the candidates. I am not an expert in assessment and was not able to fully understand the methodology the authors describe. If I have understood correctly, each item may have to be administered to a large number of candidates to calculate the relative difficulty. The other three variables seem to be easier to work out. The examples provided by the authors help to clarify the interpretation of GIMs. Many medical school use examination software to administer exams. I have a few questions for the authors. Is there any software available to create GIMs? Can GIMs be also used for summative exams at individual institutions? Or is the process too labor intensive? I find GIMs a good way to provide feedback to students without compromising the integrity of individual items.

Reviewer Expertise:

NA

No decision status is available

# Reviewer report: Reviewer response for version 1

**DOI:** 10.21956/mep.19583.r29066

**Published:** 2018-06-03

**Authors:** Tripti Srivastava

**Affiliations:** 1 Datta Meghe Institute of Medical Sciences Deemed University

This review has been migrated. The reviewer awarded 3 stars out of 5 A new and interesting concept - Graphical Item maps (GIM). The authors have adequately rationalised the need for such maps, enabling a more specific feedback that elaborates on the sub-areas of strengths and weaknesses within a subject in Multiple choice question based assessments. Details regarding measurement of relative difficulty of each item by logits from Rasch scaling would have been more helpful. It will be prudent to trial this mapping more scientifically with an estimated sample and analyse the outcome. Student perceptions about this feedback approach is a major aspect for them to actually work upon the weak areas as identified in the graph. The limitations of the study are well founded. Hope to read more about this in future.

Reviewer Expertise:

NA

No decision status is available
